# MicroRNAs signatures as potential molecular markers in mild cognitive impairment: a meta-analysis

**DOI:** 10.3389/fnagi.2024.1524622

**Published:** 2025-01-15

**Authors:** Natalia Vargas-Rondón, Yeimy González-Giraldo, Ángela Y. García Fonseca, Janneth Gonzalez, Andrés Felipe Aristizabal-Pachon

**Affiliations:** Experimental and Computational Biochemistry, Department of Nutrition and Biochemistry, Faculty of Sciences, Pontificia Universidad Javeriana, Bogotá, Colombia

**Keywords:** biomarkers, early diagnosis, mild cognitive impairment, miRNAs, neurodegeneration

## Abstract

Mild cognitive impairment (MCI) is characterized by a decline in cognitive functioning without significant interference in daily activities. Its high heterogeneity and elevated conversion rate to dementia pose challenges for accurate diagnosis and monitoring, highlighting the urgent need to identify methodologies focused on the early detection and intervention of MCI. Due to their biological characteristics, microRNAs (miRNAs) are potential candidates as non-invasive molecular markers for the identification and assessment of MCI progression. Therefore, in this study, we conducted a meta-analysis to identify the miRNAs commonly deregulated in MCI, focusing on expression profiles in plasma, serum, and extracellular vesicle samples. Our analysis identified eight upregulated miRNAs, including hsa-miR-149-3p, and four downregulated miRNAs, such as Let-7f-5p. Notably, hsa-miR-149-3p emerged as a central node in interaction networks, suggesting its crucial role in regulating cellular processes relevant to MCI. Additionally, pathway analysis revealed significant enrichment in biological processes associated with transcriptional regulation and neurodegeneration. Our results underscore the potential of circulating miRNAs as non-invasive molecular markers for MCI and open the possibility for new methodologies that enable more accurate diagnosis and monitoring of disease progression. Validating the expression of miRNAs such as hsa-miR-149-3p and Let-7f-5p, along with identifying their functional role in the specific context of MCI, is essential to establish their biological relevance. This work contributes to the understanding of the miRNA profile in mild cognitive impairment using easily accessible samples, which could be useful for the development of various strategies aimed at preventing or delaying MCI in individuals at risk of developing dementia, including Alzheimer’s disease.

## 1 Introduction

Mild Cognitive Impairment (MCI), currently termed as mild neurocognitive disorder by the Diagnostic and Statistical Manual of Mental Disorders-5 (DSM-5), is defined as a decline in cognitive functioning. MCI can be classified based on whether memory is impaired or not, as amnestic, and non-amnestic subtypes, respectively. Moreover, depending on the number of affected domains, MCI can be categorized as single or multiple domains. Other relevant characteristic is that MCI does not interfere in the daily activities (Sachs-Ericsson and Blazer, 2015). Although MCI is not a disease, it has garnered significant interest in recent years because it falls between normal aging and the early stages of dementia (Morris, 2005). It has been reported that the transition from MCI to dementia occurs at an annual rate of 12% in the general population, reaching up to 20% in higher-risk groups (Plassman et al., 2008; Petersen, 2006). This suggests that early identification and timely treatment of MCI may delay the onset of dementia. However, MCI is clinically heterogeneous, which has hindered efforts to identify and monitor it (Sabbagh et al., 2020).

The MCI identification is established considering previous cognitive functioning before memory complain onset, educational level, lifestyle, and ethnic differences (Kouzuki et al., 2022). Currently, the assessment of MCI relies on the sensitivity and specificity of cognitive and functional tests, such as the Mini-Mental Status Examination (MMSE), the Montreal Cognitive Assessment (MoCA), among others (Jia et al., 2021). However, despite their general utility, diagnostic methods for MCI are constantly evolving and not well standardized, which may lead to variations in results and a lack of consistency in the early and accurate diagnosis of MCI (Chen et al., 2021; Lee, 2023; López and Altuna, 2023).

Although molecular markers are not currently included in the diagnostic criteria (Dunne et al., 2021), their potential in identifying and assessing the risk of progression from MCI to dementia has captured the attention of medical research. Indeed, various biomarkers in cerebrospinal fluid (CSF), such as Aβ42 and tau protein, have been explored with the purpose of using them for differential diagnosis between MCI, Alzheimer’s disease (AD), and cognitively normal subjects (Dunne et al., 2021; Papaliagkas et al., 2023). However, assessing biomarkers in CSF involves an invasive procedure, thus there is an urgent need to identify more accessible and less invasive molecular markers present in peripheral fluids that allow for timely diagnosis of MCI (Álvarez-Sánchez et al., 2023; Hansson et al., 2010).

In this context, considerable attention has been directed toward microRNAs (miRNAs) as potential molecular markers for MCI. miRNAs are non-coding RNA molecules that play a crucial role in regulating gene expression (Ranganathan and Sivasankar, 2014; Ratti et al., 2020). These small molecules can repress specific genes by binding complementarily to the 3′ untranslated region (3’UTR) of mRNA, leading to either degradation or inhibition of translation (McNeill and Van Vactor, 2012). Interestingly, miRNAs have also been found to interact with other regions of mRNAs, including the 5’ UTR, the coding sequence, and gene promoters, where they can promote protein translation (Groth et al., 2007; Ørom et al., 2008). This versatility underscores their significance as regulators of cellular activity in both pathological and non-pathological conditions (O’Brien et al., 2018; Ranganathan and Sivasankar, 2014).

Recent studies have indicated that alterations in the expression of specific miRNAs may be linked to the regulation of biological processes involved in neurodegenerative diseases (Li et al., 2023; Mu et al., 2024). Furthermore, various investigations have shown that circulating miRNAs are present in peripheral fluids such as blood, serum, plasma, and saliva, as well as in extracellular vesicles like exosomes. These miRNAs can be easily detected and, due to their high stability, reflect subtle changes that occur during the early stages of neurodegeneration (Kamal and Shahidan, 2020; Kumar and Reddy, 2016).

To date, various studies have evaluated the expression of miRNAs in MCI. However, challenges exist in identifying reliable molecular markers mainly due to the lack of multi-platform standardization, the continuous discovery of new miRNAs, and the small sample size (Condrat et al., 2020). Therefore, integrative approaches such as meta-analysis, which enable the combination of data from multiple studies to identify the most relevant miRNAs, have emerged as a promising option. These methods not only contribute to increasing predictive capacity but also help overcome potential contradictions present in individual research (Roointan et al., 2023; Yuen et al., 2021).

Considering the above, our aim was to identify commonly dysregulated miRNAs in MCI and characterize their biological significance. To achieve this, we conducted a meta-analysis encompassing previously published studies on miRNA expression profiles in samples of plasma, serum, and extracellular vesicles. Additionally, we aimed to characterize the biological significance of these findings through pathway analysis and functional enrichment, as well as the construction of interaction networks between miRNAs and their target genes. This approach significantly contributes to understanding the underlying molecular mechanisms in MCI, as well as to identifying potential molecular markers for this condition.

## 2 Methods

### 2.1 Search strategy

In this work, the recommendations of the PRISMA statement for the conduct and reporting of meta-analyses were considered (Moher et al., 2009). The databases used to perform the search for original studies were PubMed, Gene Expression Omnibus (GEO), and Sequence Read Archive (SRA). The following search terms were implemented: (“miRNA”) OR (“miRNA*”) OR (“microRNA”) OR (“microRNAs”) AND (“plasma”) OR (“blood”) OR (“serum”) OR (“extracellular vesicles”) AND (“mild cognitive impairment”) OR (“MCI”) OR (“Cognitive Impairment, Mild”) OR (“Impairment, Mild Cognitive”) OR (“Cognitive Impairment*”) AND (“next generation sequencing”) OR (“NGS”) OR (“microArray”) OR (“Array”); these terms were applied in the titles, abstracts, and keywords present in the selected databases.

### 2.2 Study selection/inclusion and exclusion criteria

Original articles meeting the following criteria were included: (a) primary studies. (b) Works evaluating the differential expression profile of miRNAs in samples of plasma, serum, and/or extracellular vesicles from patients with MCI and controls. (c) Studies assessing the relative expression of miRNAs using miRNA microarrays, RNA sequencing, or qRT-PCR. (d) Studies providing a list of differentially expressed miRNAs (DE-miRNAs) or presenting raw data. (e) Studies reporting cut-off criteria for DE-miRNAs. (f) Studies reporting sample size. In contrast, studies were excluded from this analysis if: (a) they were conducted in cell lines and animal models. (b) They evaluated a limited set of pre-selected miRNAs. (c) They did not include MCI cases; they were reviews or other meta-analyses. Two investigators performed the search in an independent manner (YG-G and NV-R) until June 2023.

### 2.3 Data extraction and processing

Lists of DE-miRNAs, along with corresponding Fold-Change (FC) values and *p*-values, were obtained from publications, supplementary web pages, analysis of raw data or files provided by the authors (Kumar et al., 2017; Sun et al., 2023; Tatara et al., 2023).

For analysis of raw data, the processing of available datasets in the GEO and SRA databases was carried out as follows: studies presenting data from microarray platform (GSE147232, GSE120584) (He et al., 2021; Tao et al., 2020) and PCR panels (GSE90828) (Kayano et al., 2016), were individually processed using the GEO2R tool on the GEO NCBI, which implements the Rstudio packages GEOquery and limma from the Bioconductor project to determine DE-miRNAs (Ritchie et al., 2015). On the other hand, the Galaxy platform was utilized to determine miRNAs from studies presenting raw data available on the SRA platform (ERP133592/E-MTAB-11222) (Xiaohuan, 2022). Briefly, data in its raw form were evaluated using the FASTQ format. The Trim Galore tool was used to remove adapter sequences and non-specific dimers through read trimming and filtering. Read alignment was performed using the MiRDeep2 Mapper and MiRDeep2 Quantifier tools, taking into account human miRNA precursors reported in mirBase. Finally, the DESeq2 tool was used to determine DE-miRNAs (Mohanan et al., 2023).

In all cases, the miRNA expression profile was compared using only samples from patients with MCI and control samples. Independent lists were generated for each study, and significant miRNAs detected were organized into upregulated and downregulated categories. MiRNAs with |log 2 FC| > 0 and *p*-value <0.05 were considered DE-miRNAs. The miRBase database was used to standardize miRNA names (Griffiths-Jones et al., 2006; Roointan et al., 2023). Non-human miRNA probes were excluded from the meta-analysis.

### 2.4 Meta-analysis

The meta-analysis was conducted using the Robust Rank Aggregation (RRA) method (Võsa et al., 2014) and the Vote Counting method (Griffith et al., 2006), as described below.

#### 2.4.1 Robust rank aggregation

The Robust Rank Aggregation Approach is based on the idea of combining individual rankings from multiple studies in a solid and reliable manner. The main objective is to obtain a global ranking that reflects the hierarchy of the evaluated miRNAs, taking into account the uncertainty and potential sources of variability or bias present in the data (Kolde et al., 2012). Lists of upregulated and downregulated DE-miRNAs were separately ranked based on the relative expression changes reported in each study. The RobustRankAggreg package v1.2.1 in Rstudio was used to integrate the results from the selected studies and determine the DE-miRNAs among them (Võsa et al., 2014). The *p*-value of each miRNA indicated its ranking in the final list, and miRNAs with a p-value <0.05 were considered significant.

#### 2.4.2 Vote counting

A list was constructed containing the upregulated and downregulated DE-miRNAs from each individual study. The Vote Counting method (VCR) for ranking potential molecular biomarkers was adopted for the meta-analysis (Chan et al., 2008; Griffith et al., 2006). This methodology is based on the number of studies reporting the differential expression of a miRNA, applying a classification based on the total sample size and the Fold Change (FC) (Griffith et al., 2006). MiRNAs with consistent regulation in at least two or more studies were considered commonly dysregulated. All analyses were performed using Rstudio v4.2.3.

### 2.5 Functional enrichment analysis

The miRNAs resulting from the two meta-analysis methods were termed MCI-miRNA meta-signatures. The online tool DIANA-mirPath v.3 was employed to identify the Kyoto Encyclopedia of Genes and Genomes (KEGG) pathways and gene ontology (GO) terms potentially affected by the dysregulation of these miRNAs (Vlachos et al., 2015). This involved the exploring of various functional categories, including molecular functions, biological processes, and cellular components within the GO. Analyses were conducted separately for miRNAs showing consistent upregulation and those showing consistent downregulation. Statistical significance was assessed using the hypergeometric distribution, with the Benjamini-Hochberg false discovery rate (FDR) of 0.05 applied for multiple testing correction.

### 2.6 Construction of interaction networks of MCI-miRNAs and target genes

The interaction networks for consistently upregulated and downregulated miRNAs were constructed independently. The interaction analysis and network construction between MCI-miRNAs and their target genes were conducted using the miRNet v2.0 platform^1^. The identification of interactions was based on the miRTarBase v8.0 database, which provides curated information on miRNA-target gene interactions (Chang et al., 2020). For the network of consistently upregulated miRNAs, a filter based on betweenness centrality with a threshold of 10 was applied, allowing the analysis to focus on the most relevant nodes within the network.

### 2.7 Validation of MCI-miRNA-gene interactions using an external dataset in amnestic MCI

To validate the interactions between the MCI-miRNAs and their target genes, an external dataset of differentially downregulated genes in peripheral blood from patients with amnestic MCI was used (Bharthur Sanjay et al., 2022). The MCI-miRNAs consistently upregulated in our study were used to construct the interaction network with these genes. The network was built using the miRNet v2.0 platform (see Footnote ^1^1^^).

## 3 Results

### 3.1 Meta-analysis of miRNA expression in MCI

**Figure 1:**
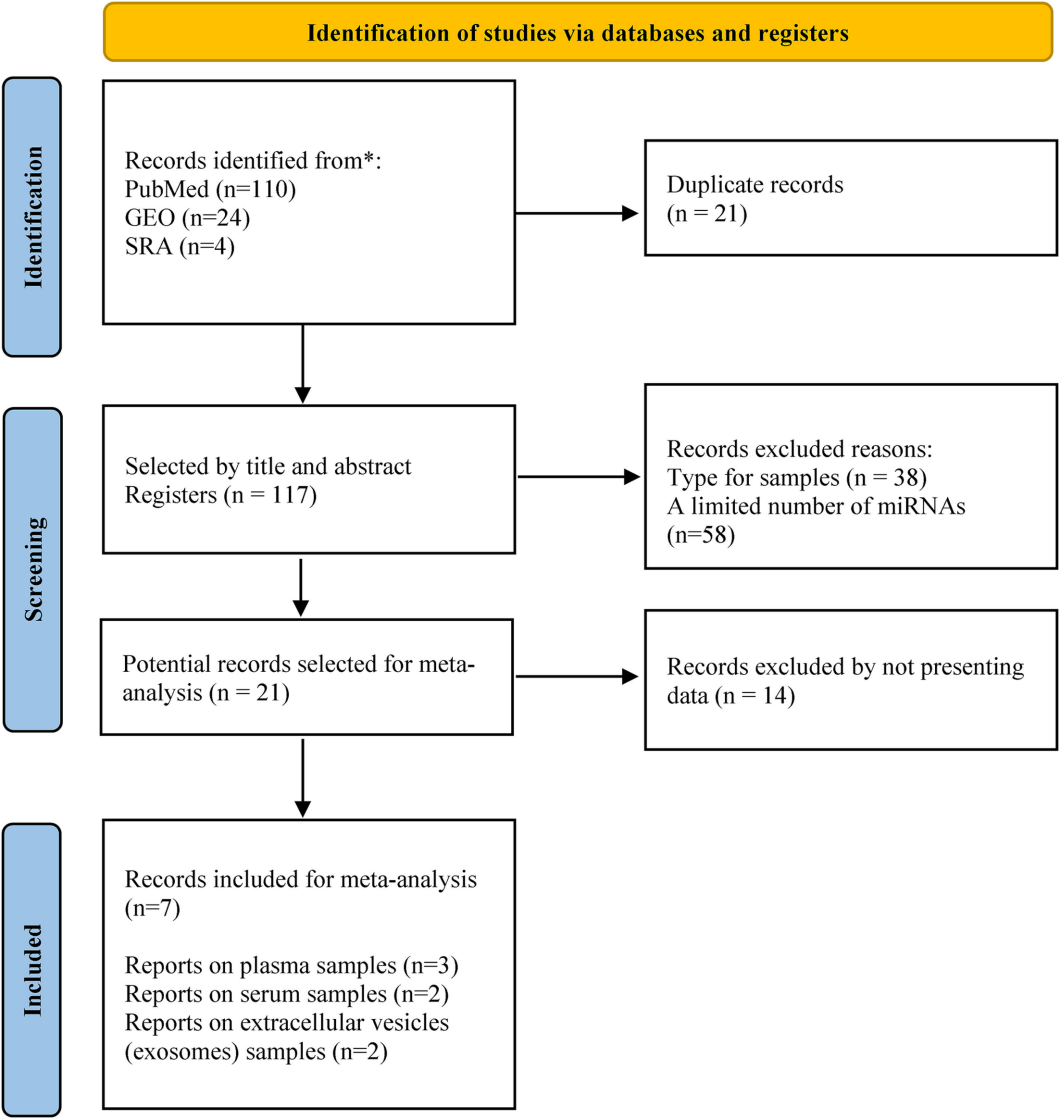
PRISMA flow diagram for the selected studies.

A total of 138 studies evaluating miRNA expression in subjects with Mild Cognitive Impairment (MCI) were identified. However, raw data or lists of differentially expressed miRNAs were not available in some cases, and others did not meet the inclusion criteria. Therefore, only seven studies were included in the final analysis. The miRNAs assessed in these studies were detected in different samples, which included plasma (3 studies), serum (2 studies), and exosomes (2 studies). The flow diagram showing the different phases for choosing the included studies is illustrated in Figure 1. Details of the characteristics of the included studies are shown in Table 1.

**Table 1:** Characteristics of included studies in the meta-analysis.

	ID dataset	Sample	Methodology	Platform	Sample size	
					Cases	Controls
He et al. (2021)	GSE147232	Plasma	Microarray	Exiqon miRCURY LNA microRNA array, 7th generation	5	5
Kayano et al. (2016)	GSE90828	Plasma	RT-PCR	Exiqon Human PCR panels I + II, V2	23	30
Kumar et al. (2017)		Serum	Microarray	Affymetrix GeneChip miRNA array V.4.0	16	14
Xiaohuan (2022)	ERP133592/E-MTAB-11222	Plasma exosomes	NGS	Illumina Hiseq 2,500	3	3
Tao et al. (2020)	GSE120584	Serum	Microarray	3D-Gene Human miRNA V21_1.0.0	32	32
Tatara et al. (2023)		Plasma	Microarray	Agilent SureScan Microarray Scanner	12	12
Sun et al. (2023)		Plasma exosomes	NGS	BGISEQ-500 platform (BGI)	48	62

This meta-analysis included 139 samples from subjects diagnosed with MCI (40 from plasma, 48 from serum, and 51 from exosomes) and 158 samples from subjects with normal cognition (47 from plasma, 46 from serum, and 65 from exosomes). A total of 640 DE-miRNAs (|log 2 FC| > 0 and *p*-value <0.05) were identified between cases and controls. After to implement the Robust Rank Aggregation (RRA) method no statistically significant results were obtained.

**Figure 2:**
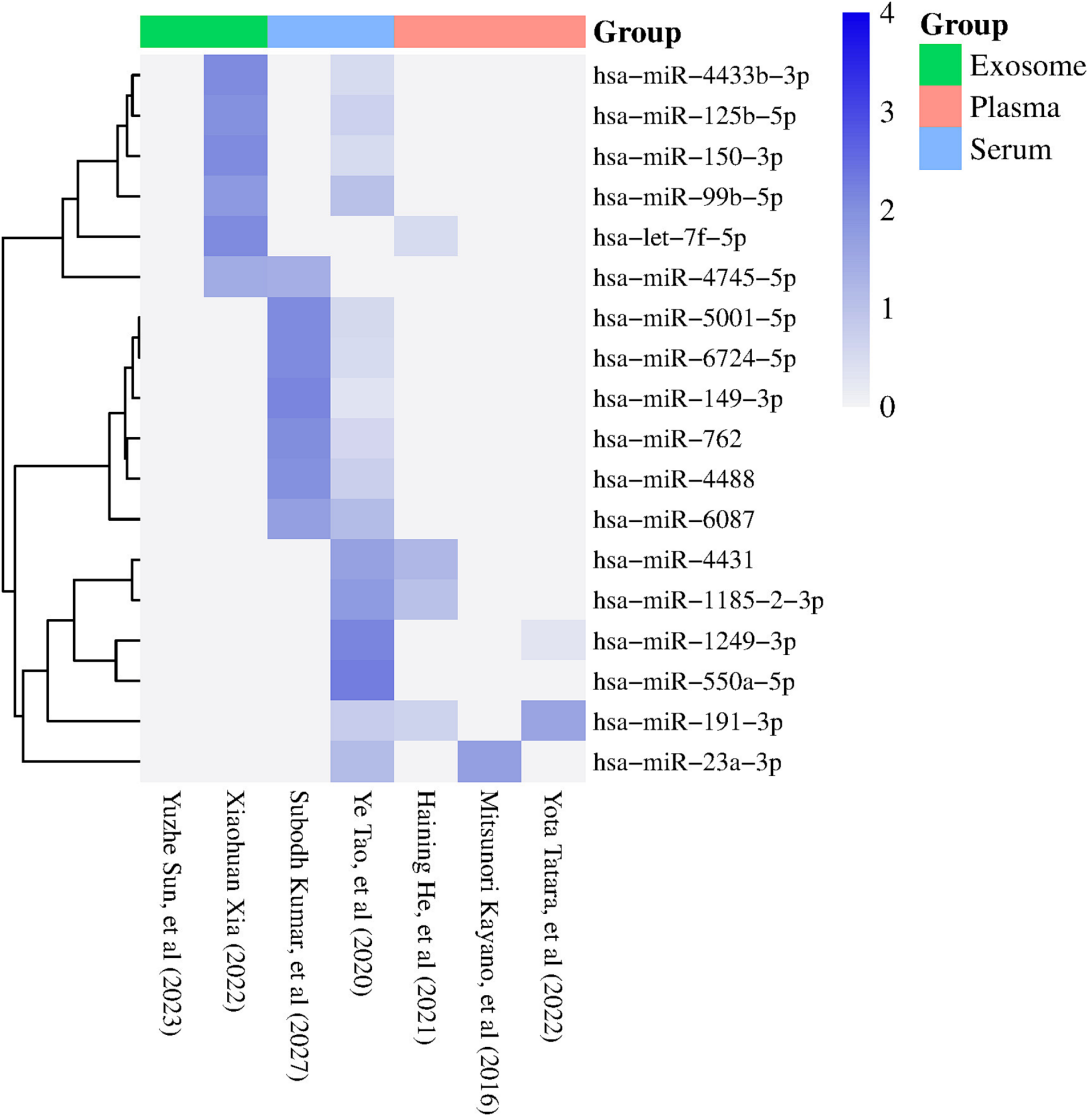
Heatmap showing the miRNAs reported in two or more studies with statistical significance (*p* < 0.05). The columns represent the studies included in the meta-analysis, while the rows correspond to the identified miRNAs. The color of the rectangles indicates the expression levels of the miRNAs, with dark blue indicating positive regulation (FC > 1) and white indicating negative regulation (FC < 1). The sample sources used in each study are specified at the top of the figure (Exosome, plasma, serum) (visual representation generated with SRPLOT, https://www.bioinformatics.com.cn/en).

According to the VCR analysis, from 64 miRNAs reported in at least two studies, 12 miRNAs showed a consistent direction of regulation among the analyzed samples. The miRNAs hsa-miR-23a-3p, hsa-miR-5001-5p, hsa-miR-6087, hsa-miR-4488, hsa-miR-6724-5p, hsa-miR-149-3p, hsa-miR-150-3p, and hsa-miR-99b-5p showed consistent upregulation, while hsa-miR-4431, hsa-miR-1185-2-3p, let-7f-5p, and hsa-miR-550a-5p showed consistent downregulation (Figure 2). Furthermore, we also observed that other miRNAs, such as hsa-miR-4745-5p, hsa-miR-4433b-3p, hsa-miR-125b-5p, hsa-miR-191-3p, hsa-miR-762, and hsa-miR-1249-3p, exceeded the identification threshold but showed inconsistent regulation patterns among samples (Figure 2). miRNAs that did not meet the identification threshold or presented inconsistent regulation patterns were excluded from further analysis (Supplementary Table S1).

### 3.2 Identification of key regulatory pathways associated with miRNAs in MCI

**Figure 3:**
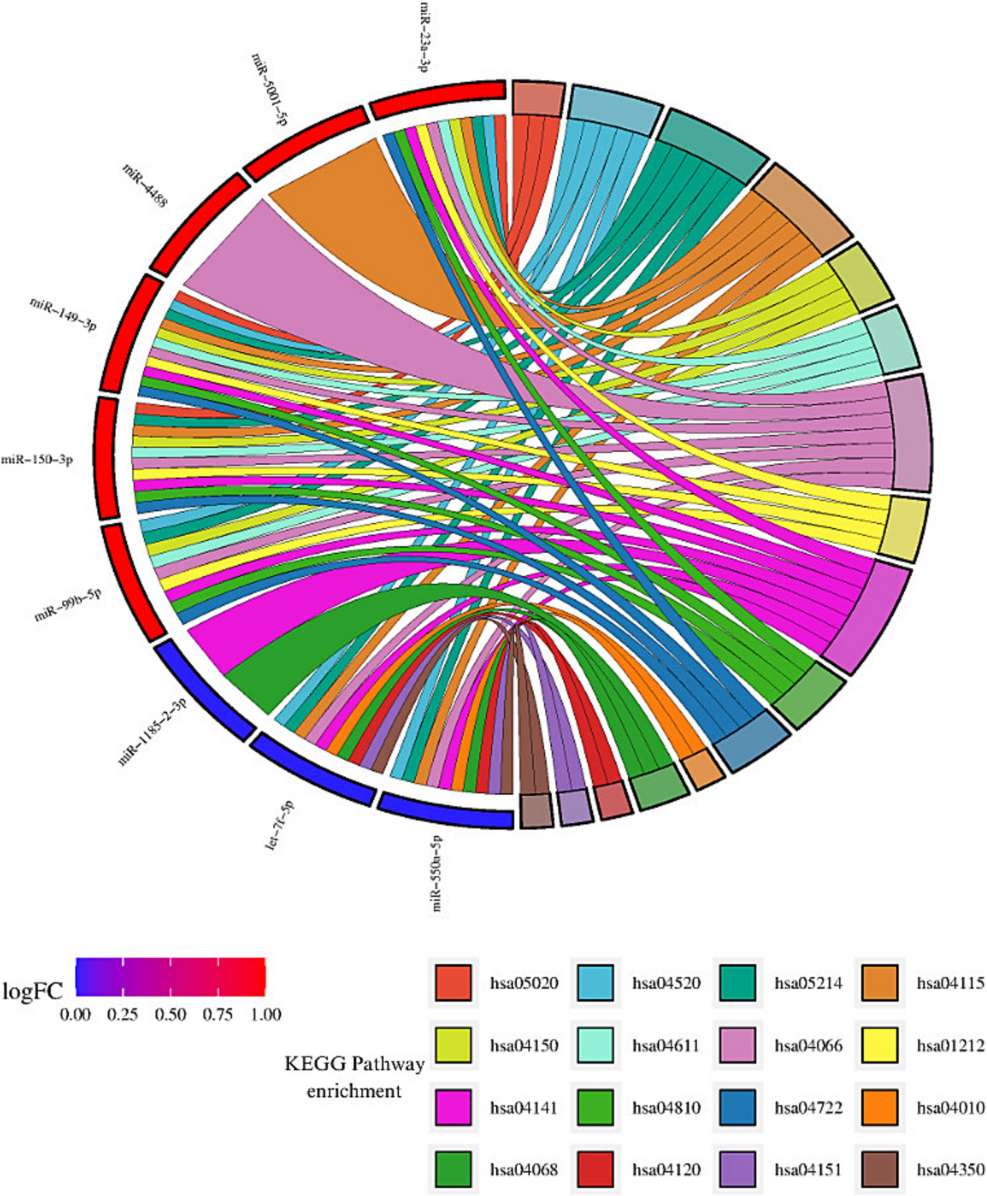
Enrichment analysis of KEGG pathway for consistent dysregulated miRNA. Sankey diagram illustrating the interaction between miRNAs and the identified KEGG pathways. miRNAs consistently upregulated are shown in red, while miRNAs consistently downregulated are depicted in blue. Each ribbon corresponds to a KEGG pathway, and the interactions or involvement of the miRNAs with each pathway are represented by the connections between the ribbons and miRNAs. miRNAs that interact with a greater number of pathways are considered the most influential in the analyzed pathways, highlighting their relevance in the regulation of key biological processes in MCI. The identified pathways include: Prion diseases (hsa05020), Adherens junction (hsa04520), Glioma (hsa05214), p53 signaling pathway (hsa04115), mTOR signaling pathway (hsa04150), Platelet activation (hsa04611), HIF-1 signaling pathway (hsa04066), Focal adhesion (hsa01212), Protein processing in endoplasmic reticulum (hsa04141), Regulation of actin cytoskeleton (hsa04810), Neurotrophin signaling pathway (hsa04722), MAPK signaling pathway (hsa04010), FoxO signaling pathway (hsa04068), Ubiquitin mediated proteolysis (hsa04120), PI3K-Akt signaling pathway (hsa04151), and TGF-beta signaling pathway (hsa04350) (visual representation generated with SRPLOT, https://www.bioinformatics.com.cn/en).

The enrichment analysis of KEGG and GO terms for miRNAs with consistent upregulation and downregulation was performed independently. For miRNAs showing consistent upregulation (hsa-miR-23a-3p, hsa-miR-5001-5p, hsa-miR-6087, hsa-miR-4488, hsa-miR-6724-5p, hsa-miR-149-3p, hsa-miR-150-3p, and hsa-miR-99b-5p, a total of 34), significantly enriched pathways were identified. Among these, the HIF-1 signaling pathway (hsa04066), mTOR signaling pathway (hsa04150), platelet activation (hsa04611), p53 signaling pathway (hsa04115), regulation of actin cytoskeleton (hsa04810), protein processing in endoplasmic reticulum (hsa04141) and neurotrophin signaling pathway (hsa04722) were regulated by four or more miRNAs. We focused on those pathways based on their important role in MCI, according to previous reports previously detected with MCI (Figure 3).

Regarding miRNAs exhibiting consistent downregulation (hsa-miR-4431, hsa-miR-1185-2-3p, let-7f-5p, and hsa-miR-550a-5p), a total of 35 significantly enriched pathways were identified. Some of these pathways were the following: the FoxO signaling pathway (hsa04068) and protein processing in the endoplasmic reticulum (hsa04141) were regulated by three or more miRNAs (Table 2). Additionally, pathways related to the hippo signaling pathway (hsa04390), ubiquitin-mediated proteolysis (hsa04120), PI3K-Akt signaling pathway (hsa04115), MAPK signaling pathway (hsa04010), and TGF-beta signaling pathway (hsa04350) were associated with genes modulated by these miRNAs (Figure 3).

**Table 2:** Kyoto Encyclopedia of Genes and Genomes (KEGG) pathway enrichment by consistently regulated miRNAs according to the number of miRNAs involved in the enrichment.

KEGG pathway ID	Name KEGG pathway	#miRNAs	#Genes	*p*-value	miRNAs regulation
hsa04115	p53 signaling pathway	4	21	0,002511395	Consistent upregulation
hsa04150	mTOR signaling pathway	4	19	0,007946105	
hsa04611	Platelet activation	4	30	0,007946105	
hsa04066	HIF-1 signaling pathway	5	27	0,010101988	
hsa01212	Focal adhesion	4	43	0,028135459	
hsa04141	Protein processing in endoplasmic reticulum	4	35	0,029621332	
hsa04810	Regulation of actin cytoskeleton	4	42	0,032199958	
hsa04722	Neurotrophin signaling pathway	4	26	0,044696914	
hsa04141	Protein processing in endoplasmic reticulum	3	42	8,90E-05	Consistent downregulation
hsa04068	FoxO signaling pathway	3	34	0,00421181	

The GO terms analyzed in the enrichment of the positively and negatively regulated miRNA groups showed significant results (Supplementary Table S2). It is noteworthy that both upregulated and downregulated miRNAs are involved in a variety of biological processes, cellular components, and molecular functions that may be closely related to MCI.

**Figure 4:**
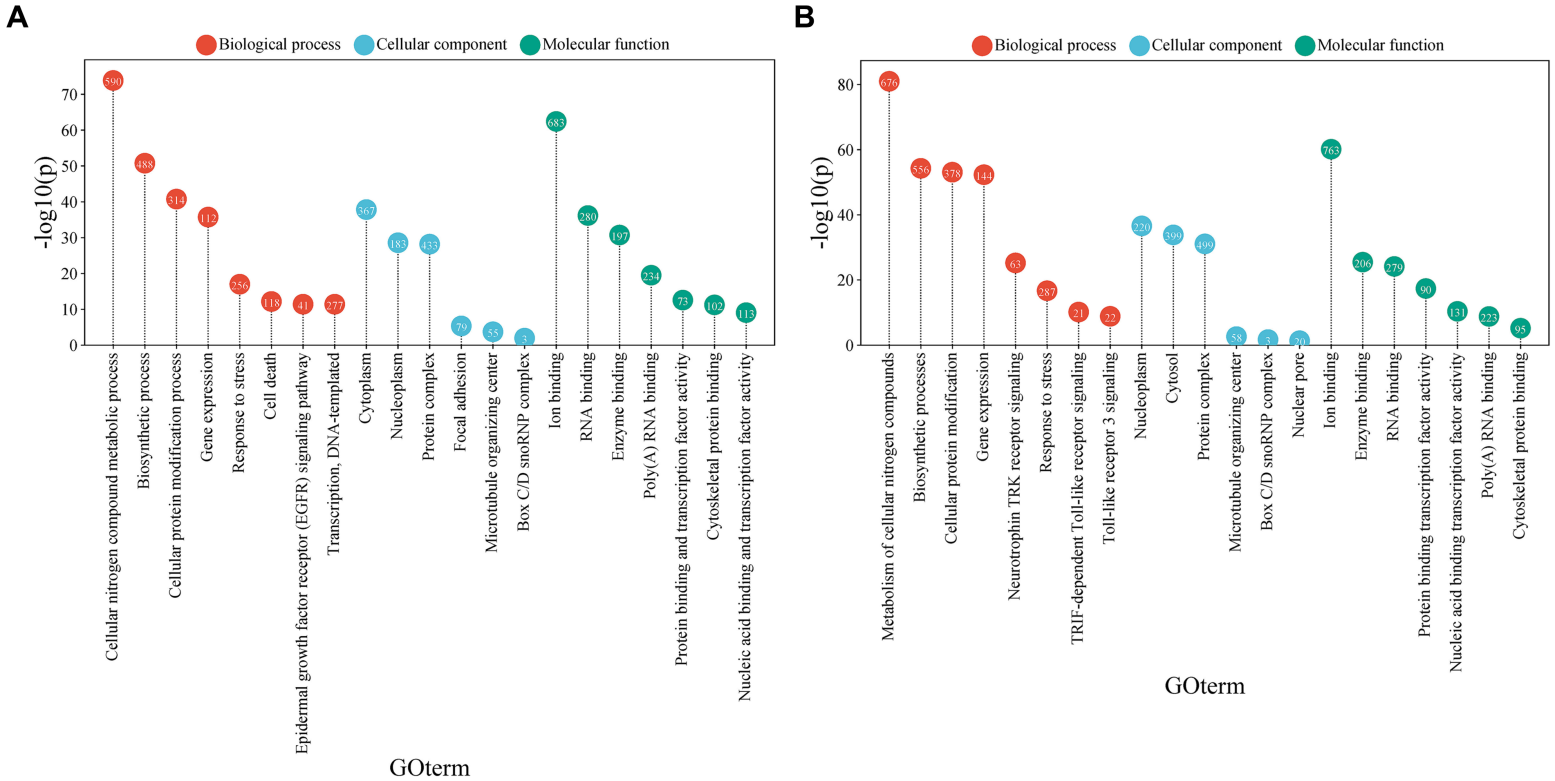
Gene ontology (GO) term analysis associated with biological functions, cellular processes, or cellular components related to miRNAs. **(A)** GO terms associated with miRNAs that are consistently upregulated. **(B)** GO terms associated with miRNAs that are consistently downregulated. The size of each circle represents the proportion of genes associated with the corresponding GO term. The *X*-axis shows the GO terms, while the *Y*-axis reflects the -log10(*p*-value), indicating the statistical significance of the association between miRNAs and the GO terms (visual representation generated with SRPLOT, https://www.bioinformatics.com.cn/en).

Regarding biological processes, both miRNA groups showed a strong association with the metabolism of cellular nitrogen compounds and biosynthetic processes, as well as with protein modification and gene expression regulation. The observed differences between upregulated and downregulated miRNAs provide further insights into their potential roles in MCI. Upregulated miRNAs showed a stronger association with the stress response and cell death (Figure 4A). In contrast, downregulated miRNAs were more strongly associated with the signaling of neurotrophin TRK receptors and Toll-like receptor signaling pathways, such as the TRIF-dependent pathway and Toll-like receptor 3 signaling (Figure 4B).

Regarding molecular functions, both miRNA groups showed a notable association with ion binding and RNA binding. However, upregulated miRNAs were more linked to protein binding and transcription factor activity.

### 3.3 Network analysis reveals key regulatory nodes among miRNAs and target genes in MCI

The miRNAs that showed consistent regulation across different samples of subjects with MCI and controls were used to build a network to determine their potential interactions with target genes. miRNA-mRNA interaction networks were constructed independently for miRNAs consistently upregulated and downregulated.

**Figure 5:**
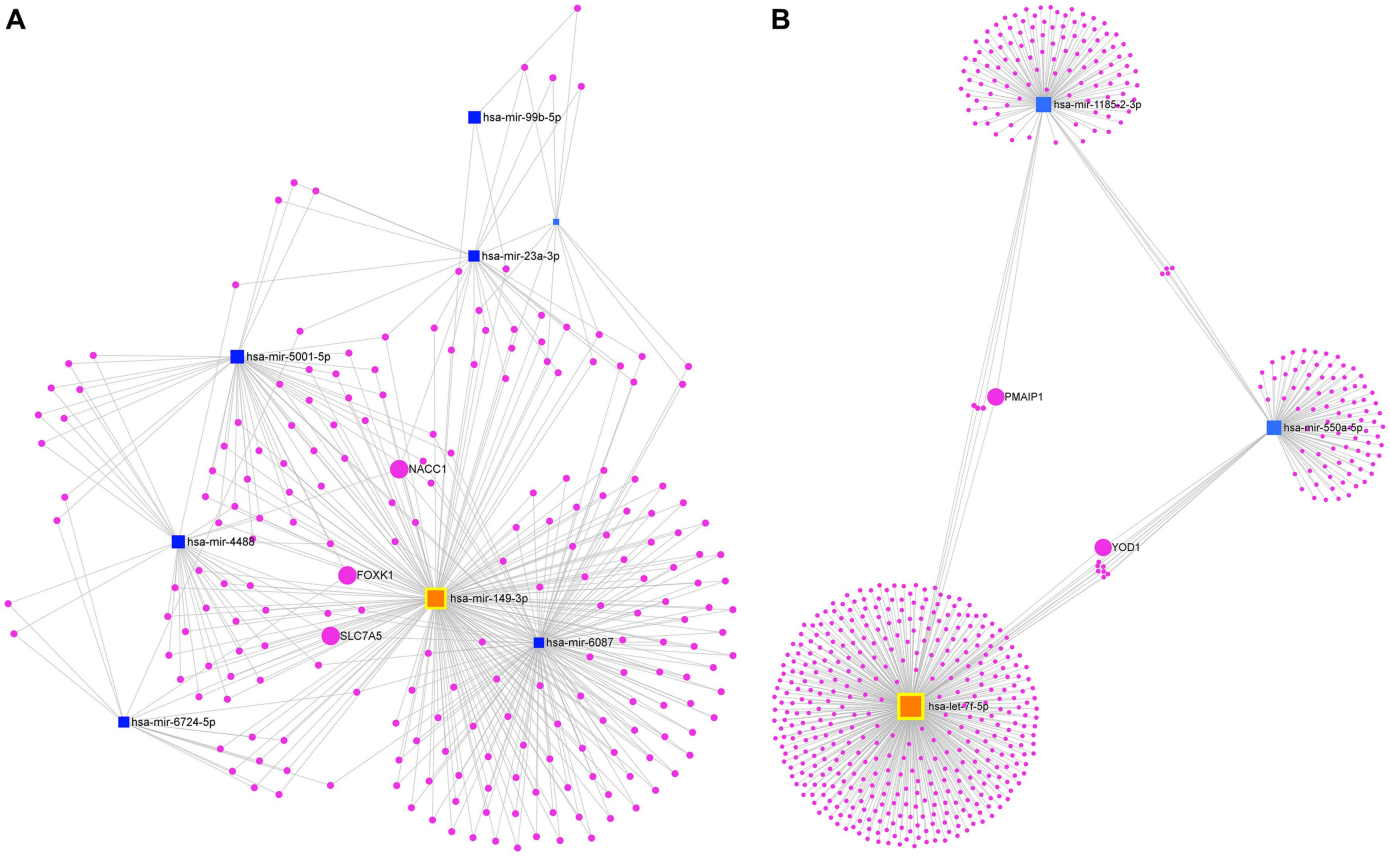
Analysis of the mRNA-miRNA interaction network. **(A)** Network of consistently upregulated miRNAs and **(B)** Network of consistently downregulated miRNAs. Blue squares represent miRNAs, while fuchsia circles represent mRNAs. The size of the squares and circles reflects the degree of connectivity of each element. The miRNA with the highest degree of connectivity in each network is highlighted in orange.

The interaction network for the eight miRNAs consistently upregulated identified a total of 1,496 nodes and 1,716 edges. Specifically, the network comprised 8 miRNA nodes and 1,488 mRNA nodes. Each mRNA node represents a target gene regulated by one or more of the upregulated miRNAs. In this network, the hsa-miR-149-3p represented the most important node with a high degree of connectivity (Degree = 834). On the other hand, it is remarkable that this analysis shows that various of these miRNAs convergent on the same target gene, which are key for connecting this network. For instance, the following genes: *NACC1* (nucleus accumbens associated 1), *SLC7A5* (solute carrier family 7 member 5), and *FOXK1* (forkhead box K1) were identified as the most important nodes in our network based on their high degree and betweenness. Specifically, *NACC1* was found to be a common target of hsa-miR-149-3p, hsa-miR-6087, hsa-miR-23a-3p, and hsa-miR-4488. Similarly, *SLC7A5* was regulated by hsa-miR-149-3p, hsa-miR-6087, hsa-miR-4488, and hsa-miR-6724-5p, while *FOXK1* (hairpin box K1) was the target of hsa-miR-149-3p, hsa-miR-6087, hsa-miR-5001-5p, and hsa-miR-6724-5p (Figure 5A).

In contrast, the first-order interaction network of the four consistently downregulated miRNAs revealed a total of 599 nodes and 612 edges. This network consisted of three miRNA nodes and 596 mRNA nodes. Let-7f-5p stood out as the main node in the network (Degree = 397). We identified *BACH1* (BTB domain and CNC homolog 1), *PMAIP1* (phorbol-12-myristate-13-acetate-induced protein 1), *ZNF264* (zinc finger protein 264), and *RBM12B* (RNA binding motif protein 12B) as mRNA targets commonly regulated by both let-7f-5p and hsa-miR-1185-2-3p. Additionally, *MOB4* (MOB family member 4, phocein), *WNK1* (WNK lysine deficient protein kinase 1), *KLHL15* (kelch like family member 15), and *HSPE1-MOB4* (HSPE1-MOB4 readthrough) were identified as mRNA targets shared between hsa-miR-1185-2-3p and hsa-miR-550a-5p. Furthermore, *GABPB1* (GA binding protein transcription factor subunit beta 1), *SMC1A* (structural maintenance of chromosomes 1A), *SEMA4C* (semaphorin 4C), *SLC38A7* (solute carrier family 38 member 7), *YOD1* (YOD1 deubiquitinase), *KIAA1328* (hinderin), *ZNF556* (zinc finger protein 556), and *PPP1R15B* (protein phosphatase 1 regulatory subunit 15B) were also identified as mRNA targets commonly regulated by let-7f-5p and hsa-miR-550a-5p (Figure 5B).

### 3.4 Validation of miRNA-target gene interaction: identification of key MCI-miRNAs in peripheral blood

The MCI-miRNAs with consistent upregulation were used to evaluate their interactions with differentially expressed genes in peripheral blood from patients with amnestic MCI (Bharthur Sanjay et al., 2022). The resulting interaction network included 4 miRNAs and 35 genes, with hsa-miR-23a-3p being the most prominent node, showing a connectivity (Degree = 35) (Supplementary Figure S1).

## 4 Discussion

In this work, we performed an integrative meta-analysis of miRNA expression profiles in plasma, serum, and extracellular vesicles, specifically exosomes, from subjects with MCI and control subjects. We identified eight consistently upregulated miRNAs (hsa-miR-23a-3p, hsa-miR-5001-5p, hsa-miR-6087, hsa-miR-4488, hsa-miR-6724-5p, hsa-miR-149-3p, hsa-miR-150-3p, and hsa-miR-99b-5p) and four consistently downregulated miRNAs (hsa-miR-4431, hsa-miR-1185-2-3p, let-7f-5p, and hsa-miR-550a-5p) by means of VCR method. The interaction network analysis between miRNA-mRNA highlighted the possible key role of hsa-miR-149-3p and Let-7f-5p in MCI.

The hsa-miR-149-3p was found as a central node with a high degree of connectivity in the interaction network of upregulated miRNAs (Figure 4A), suggesting its possible influence in regulating multiple cellular processes through the modulation of a wide range of target genes. Specifically, hsa-miR-149-3p was found to be upregulated in serum samples, indicating its potential as a minimally invasive diagnostic marker (Chen et al., 2008). This miRNA has been implicated in various pathological conditions, including several types of cancer such as glioma (He et al., 2018; Lu et al., 2022; Wang et al., 2023) and central nervous system diseases like amyotrophic lateral sclerosis (ALS) (Katsu et al., 2019). Additionally, it is involved in several critical biological processes such as cell proliferation, apoptosis, and cell cycle regulation (Wang et al., 2023). However, its potential influence on cognitive decline has not yet been established (Xiamin et al., 2020), and the expression of hsa-miR-149-3p in serum samples in MCI has not been validated.

Interestingly, a study reported a positive correlation between serum levels of hsa-miR-149, the precursor of hsa-miR-149-3p, and MMSE scores in Alzheimer’s disease (AD) patients, suggesting that miR-149 might be involved in the progression of this disease (Du et al., 2021). Given that the MMSE is widely used for assessing MCI, it is crucial to investigate whether this association also manifests in specific pathological processes of MCI and determine which of the two strands of the hsa-miR-149 precursor (hsa-miR-149-3p or hsa-miR-149-5p) could be specially involved in these conditions. Contrary to our observations in this meta-analysis, in AD patients this miRNA was observed downregulated. However, in another work, a similar pattern of expression was observed in patients with HIV-associated neurocognitive disorder, where the levels of hsa-miR-149-3p was found increased by comparing this group with HIV patients without this disorder (Asahchop et al., 2016). These findings support the idea that this miRNA might reflect the underlying biological alterations in neurocognitive disorders.

From the interaction network analysis in our study, we also determined that the consistently upregulated miRNAs, including hsa-miR-149-3p, exert significant influence in regulating key genes. We observed that *NACC1* and *FOXK1* were targets of hsa-miR-149-3p and proved to be important nodes (hubs) within the network. Both *NACC1* and *FOXK1* have been reported to act as transcriptional regulators (Gao et al., 2020; Schoch et al., 2017). Specifically, *NACC1* is expressed in the CNS (Mackler et al., 2000; Xie et al., 2024) and plays a crucial role in maintaining synaptic plasticity (Daniel et al., 2023; Shen et al., 2007). On the other hand, *FOXK1*, whose DNA methylation has been associated with AD, has been linked to oxidative stress (De Jager et al., 2014; Sanchez-Mut and Gräff, 2015). These associations suggest that both *NACC1* and *FOXK1* could play significant roles in various biological processes related to MCI. However, further research is needed to confirm their direct involvement and to understand in more detail the specific mechanisms through which these genes and their interactions with hsa-miR-149-3p could influence the progression and pathogenesis of MCI. Additionally, it remains to be determined whether the expression of these genes can be indicative of pathological states related to MCI.

Regarding the consistently downregulated miRNAs, the interaction network identified Let-7f-5p as a central node with a high degree of connectivity (Figure 4B), showing consistent negative regulation in plasma and plasma-derived exosomes from subjects with MCI. It is important to note that the expression of Let-7f-5p has been reported differentially across various sample types in subjects with AD. For instance, Hara et al. (2017) reported upregulation of Let-7f-5p in serum samples from AD patients, whereas Leidinger et al. (2013) reported reduced levels of Let-7f-5p in whole blood. However, a study examining the association between plasma miRNA levels and changes in cognition over time found a negative correlation between Let-7f-5p and MMSE scores, suggesting that Let-7f-5p expression is linked to cognitive decline (Comfort et al., 2022). This indicates potential variation in its expression depending on the disease stage, ranging from pre-symptomatic or MCI stages to dementia or AD development. Nevertheless, further research is needed to confirm this relationship.

The variability in Let-7f-5p expression across different sample types underscores the need to consolidate these findings to establish its utility as a potential marker for MCI. Consolidating data will enable its potential clinical use, offering a non-invasive tool for early detection of MCI and monitoring progression to dementia or AD.

The interaction network analysis in our study revealed that consistently downregulated miRNAs, including Let-7f-5p, exert significant influence in regulating key genes, prominently featuring *PMAIP1*. This gene, recognized as an essential mediator of p53-dependent apoptosis, plays a crucial role in neurodegenerative disorders, particularly in cellular stress response (Gil-Jaramillo et al., 2023; Roufayel et al., 2022). Furthermore, a recent omics data integration study identified *PMAIP1* as a potential risk gene for AD (Wang et al., 2021). These observations and the increased risk of progression to AD due to MCI open new perspectives for evaluating the possible relationship between *PMAIP1* and MCI, which could be crucial for better understanding the underlying mechanisms in the transition from MCI to AD.

Additionally, we identified hsa-miR-23a-3p as a central node in the interaction network between upregulated miRNAs and differentially expressed genes in the peripheral blood of patients with amnestic MCI, suggesting its potential as a key regulator in the pathogenesis of MCI. Previously, hsa-miR-23a-3p has been reported as downregulated in plasma extracellular vesicles and serum from patients with AD (Galimberti et al., 2014; Gámez-Valero et al., 2019). However, studies in brain tissue from AD patients have shown upregulation of this miRNA (Lau et al., 2013), highlighting a discrepancy in its expression depending on the type of sample and the biological context in which it is analyzed. According to our results, hsa-miR-23a-3p was consistently reported as upregulated in serum and plasma samples from MCI patients, suggesting that its regulation may depend on factors specific to the disease stage and the particular biological environments of each sample. This discrepancy in the regulation patterns of hsa-miR-23a-3p could indicate a complex biological function across the different stages of the disease. In this context, further studies are imperative to investigate the functional role of hsa-miR-23a-3p in greater depth, in order to elucidate its contribution to the underlying pathological mechanisms in both MCI and AD.

Our KEGG analysis revealed that HIF-1 signaling pathway, p53 signaling pathway, protein processing in the endoplasmic reticulum, and focal adhesion pathways were consistently regulated by both groups of miRNAs (Figure 3). Considering these pathways’ critical roles in neuronal function and early stages of neurodegeneration (Rønn et al., 2000; Sin and Nollen, 2015), these findings suggest that the regulation of these pathways may be linked to pathological mechanisms associated with MCI. Moreover, the involvement of miRNAs in both repression and activation of these pathways highlights the diversity of their regulatory function and suggests a complex interaction of potential molecular mechanisms underlying MCI.

Finally, we found that both groups of miRNAs were associated with biological processes that could influence the regulation of metabolic pathways essential for neuronal function. Their involvement in the modification of cellular proteins and the regulation of gene expression highlights their potential role in modulating key processes for neuronal plasticity and cognition, which are critical factors for maintaining cognitive abilities (Bhat et al., 2022).

Considering that the upregulated miRNAs showed a stronger association with the stress response and cell death, it is possible that they are involved in the adaptive response to neuronal damage, contributing to cellular defense mechanisms and participating in the regulation of neuronal homeostasis in response to stress that may be occurring during MCI (Butterfield, 2023). On the other hand, the consistent downregulation of miRNAs associated with Toll-like receptor signaling pathways suggests an alteration in immune responses and the regulation of brain inflammation. These processes have been identified as key factors in the pathogenesis of MCI, indicating that the regulation of these pathways could have important implications for modulating neuroinflammation (Liang and Wang, 2021; Su et al., 2016).

Together, these results suggest that both upregulated and downregulated miRNAs play complementary roles in the pathophysiology of MCI. Understanding how these miRNAs influence these biological processes could open new avenues for the development of therapeutic strategies aimed at restoring cellular balance and mitigating the effects of neuroinflammation in MCI.

Although the meta-analyses play a crucial role in detecting miRNAs with more reliable differential expression by integrating multiple studies, thereby increasing sample size and overcoming limitations inherent in individual studies (Wang et al., 2015):these analyses may face certain limitations, such as the scarcity of studies evaluating miRNA profiles in subjects with MCI. Additionally, the lack of availability of raw data poses another obstacle to research. For example, while we identified studies in PubMed analyzing miRNA profiles in MCI subjects, many authors do not share raw data in any database. Therefore, it is essential for all studies to provide raw data on expression profiles, which would facilitate access to and reuse of these public datasets for future analyses (Forero et al., 2019).

Other limitation related to our work was the heterogeneity between the included studies, which could affect the robustness of the results obtained using the RRA method. Differences in the platforms and protocols used, along with the scarcity of studies evaluating miRNAs in patients with MCI, complicated the interpretation of the findings. However, the complementary VCR approach provided additional insights, helping to mitigate some of these challenges. Since research on miRNAs in MCI is an emerging field, we believe that addressing these limitations in future studies is essential for identifying the profile and functional role of miRNAs in MCI.

In general, our findings are promising, as they open new perspectives for future research that could lead to the identification of specific molecular markers for MCI in easily accessible biological samples, providing valuable insights into the underlying molecular mechanisms of this condition. Validating the expression of hsa-miR-149-3p, Let-7f-5p, and hsa-miR-23a-3p in plasma and serum samples, as well as elucidating their interactions with target genes, are crucial steps to confirm their clinical relevance in the context of MCI. This knowledge could be key to developing new therapeutic strategies aimed at modulating the expression of specific miRNAs or the activity of their target genes, with the goal of preventing, delaying, or reversing cognitive decline in individuals at risk of developing AD (Abidin et al., 2023; Li et al., 2024).

The identification of specific miRNA profiles for MCI, along with a detailed understanding of the biological mechanisms involved, has crucial implications not only for the diagnosis and treatment of this condition but also for optimizing clinical management and designing more effective and personalized therapeutic strategies. This approach would represent a significant step toward precision medicine in the field of neurodegenerative diseases (Nuzziello et al., 2020). Furthermore, the detection of miRNAs in blood samples, supported by advanced technologies such as next-generation sequencing and PCR, would facilitate their integration into daily clinical practice, improving both diagnostic accuracy and therapeutic outcomes. However, for this approach to be clinically viable, it is essential to validate the findings in a broader biological context, ensuring their applicability across diverse populations and clinical settings, thus consolidating their inclusion in routine clinical practice.

In summary, our results pave the way for promising future research that could bring these advances to fruition, transforming the diagnosis and management of MCI and other neurodegenerative diseases, and providing a solid foundation for future clinical applications.

## Data Availability

The datasets presented in this study can be found in online repositories. The names of the repository/repositories and accession number(s) can be found in the article/Supplementary material.

## Footnotes

^1^ https://www.mirnet.ca/

## Supplementary material

SUPPLEMENTARY FIGURE S1 Validation of MCI-miRNA-gene interactions using an external dataset from amnestic MCI. Blue squares represent miRNAs, while fuchsia circles represent mRNAs. The size of the squares and circles reflects the connectivity degree of each element. The miRNA with the highest connectivity degree in each network is highlighted in orange (visual representation generated with SRPLOT, https://www.bioinformatics.com.cn/en).
