# Supplementary figures and images for: MicroRNAs signatures as potential molecular markers in mild cognitive impairment: a meta-analysis
Source: Front Aging Neurosci. 2025 Jan 15;16:1524622. doi: 10.3389/fnagi.2024.1524622 (PMC11774935; doi:10.3389/fnagi.2024.1524622)

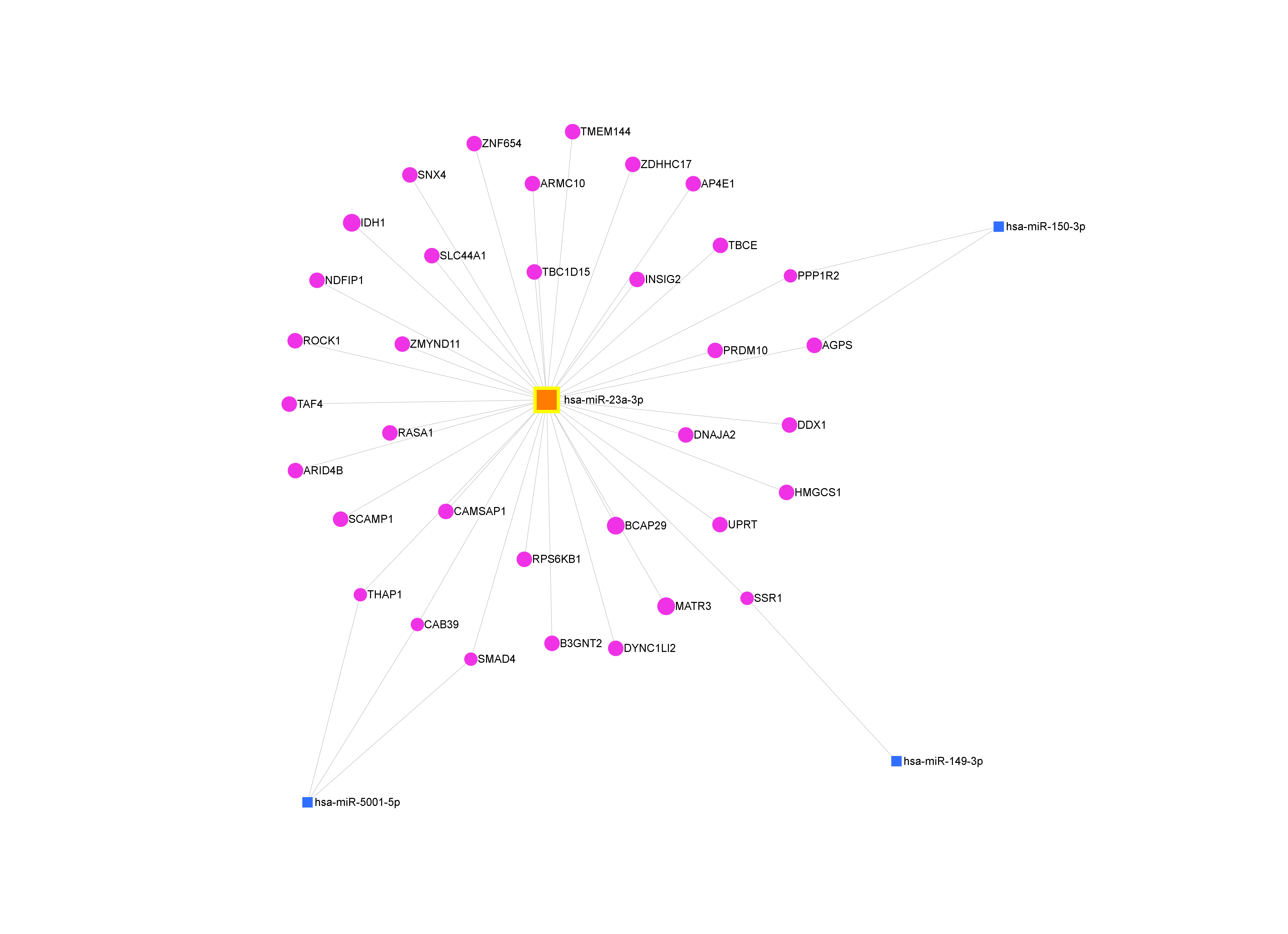

Supplement: SUPPLEMENTARY FIGURE S1 — Validation of MCI-miRNA-gene interactions using an external dataset from amnestic MCI. Blue squares represent miRNAs, while fuchsia circles represent mRNAs. The size of the squares and circles reflects the connectivity degree of each element. The miRNA with the highest connectivity degree in each network is highlighted in orange (visual representation generated with SRPLOT, https://www.bioinformatics.com.cn/en). [file Image_1.JPEG]
